# Standard breath-hold versus free-breathing real-time cine cardiac MRI—a prospective randomized comparison in patients with known or suspected cardiac disease

**DOI:** 10.1093/ehjimp/qyaf042

**Published:** 2025-04-25

**Authors:** Mohamed Elshibly, Simran Shergill, Kelly Parke, Charley Budgeon, Rachel England, Ciaran Grafton-Clarke, Fathelrahman Elshibly, Peter Kellman, Gerry P McCann, Jayanth R Arnold

**Affiliations:** Department of Cardiovascular Sciences, University of Leicester, BHF Cardiovascular Research Centre, Glenfield Hospital, Groby Road, Leicester, LE3 9QP Leicester, UK; Department of Cardiovascular Sciences, University of Leicester, BHF Cardiovascular Research Centre, Glenfield Hospital, Groby Road, Leicester, LE3 9QP Leicester, UK; Department of Cardiovascular Sciences, University of Leicester, BHF Cardiovascular Research Centre, Glenfield Hospital, Groby Road, Leicester, LE3 9QP Leicester, UK; Department of Cardiovascular Sciences, University of Leicester, BHF Cardiovascular Research Centre, Glenfield Hospital, Groby Road, Leicester, LE3 9QP Leicester, UK; Cardiovascular Epidemiology Research Centre, School of Population and Global Health, University of Western Australia, Perth, Australia; Department of Cardiovascular Sciences, University of Leicester, BHF Cardiovascular Research Centre, Glenfield Hospital, Groby Road, Leicester, LE3 9QP Leicester, UK; Department of Cardiovascular Sciences, University of Leicester, BHF Cardiovascular Research Centre, Glenfield Hospital, Groby Road, Leicester, LE3 9QP Leicester, UK; Division of General Medicine, Nottingham University Hospitals, Nottingham, UK; National Heart, Lung, and Blood Institute, National Institutes of Health, Bethesda, MD, USA; Department of Cardiovascular Sciences, University of Leicester, BHF Cardiovascular Research Centre, Glenfield Hospital, Groby Road, Leicester, LE3 9QP Leicester, UK; Department of Cardiovascular Sciences, University of Leicester, BHF Cardiovascular Research Centre, Glenfield Hospital, Groby Road, Leicester, LE3 9QP Leicester, UK

**Keywords:** cardiac magnetic resonance, CINE imaging, real-time cine, steady-state free precession

## Abstract

**Aims:**

Cardiovascular magnetic resonance (CMR) is established as the reference standard for cardiac volumetric assessment. Despite the accuracy and robustness of steady-state free precession (SSFP) cine imaging, its use may prove challenging in patients with arrhythmia and in those who cannot perform repeated breath holds. An alternative solution may be a free-breathing electrocardiogram (ECG)-triggered, retro-gated, real-time cine sequence. This study sought to compare left ventricular volumetric, wall motion, and thickness assessment with both techniques.

**Methods and results:**

Consecutive patients with known or suspected cardiac disease referred for clinical CMR were studied at 3-Tesla. Participants underwent short-axis standard SSFP and real-time cine imaging in a randomized order within the same scan. Between sequence agreement and mean difference were compared for end-diastolic volume (EDV), end-systolic volume (ESV), stroke volume, ejection fraction (EF), left ventricular mass (LVM), maximal wall thickness (MWT), and wall motion score index (WMSi). Two hundred and two patients (mean age 61 ± 14 years, 51% male and 14% irregular rhythm) were studied. All left ventricular indices showed good–excellent agreement between the two methods [intraclass correlation coefficient (95% confidence interval), EDV 0.96 (0.95–0.97), ESV 0.96 (0.94–0.97), EF 0.85 (0.81–0.88), LVM 0.93 (0.91–0.95), MWT 0.80 (0.75–0.85), and WMSi 0.93 (0.91–0.95)].

**Conclusion:**

In patients with known or suspected cardiac disease, real-time cine imaging demonstrates good–excellent reproducibility of LV volumetric, wall thickness and resting wall motion assessment when compared with standard SSFP (Trial registration: NCT05221853).

## Graphical Abstract

**Graphical Abstract:**
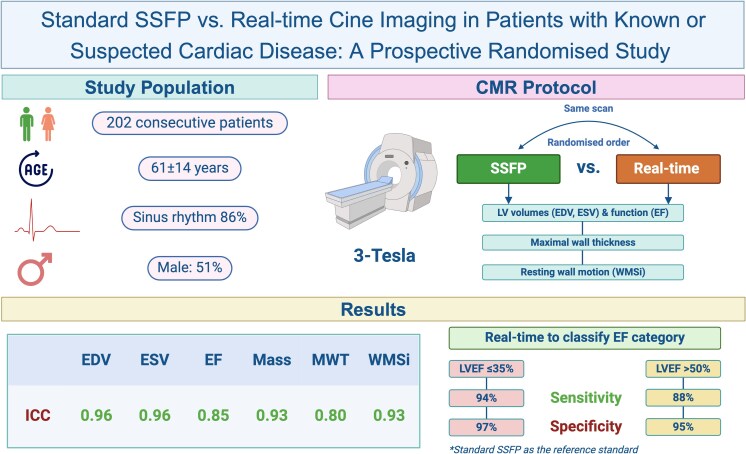

## Introduction

Cardiovascular magnetic resonance (CMR) cine imaging is the gold-standard for assessing left ventricular (LV) volumes, systolic function, and mass.^1^ The preferred technique is an electrocardiogram (ECG)-gated, breath-hold, steady-state free precession (SSFP) pulse sequence, with 10–15 contiguous short-axis (SAX) images acquired over 5–8 min.^2,3^ SSFP imaging enables accurate quantification of LV systolic function—a powerful predictor of long-term survival and key determinant for therapeutic interventions such as drug and device therapy.^4,5^ Furthermore, CMR affords improved visualization of wall motion abnormalities^6^ and accurate assessment of wall thickness, increasing the confidence to diagnose coronary artery disease or allow appropriate risk stratification in hypertrophic cardiomyopathy.^7–9^

However, standard SSFP cine imaging is time-consuming, requiring multiple sequential breath-holds, which may be challenging or tiring for some patients. Furthermore, image quality may be degraded in those with arrhythmia. These difficulties may be circumvented with a free-breathing, ECG-triggered, real-time imaging sequence,^10^ allowing a complete dataset to be acquired rapidly within 1 min. This not only improves time efficiency and patient tolerability but non-rigid motion correction yields a single image series with high temporal and spatial resolution and provides augmented robustness in patients with arrhythmia.^11,12^

Nonetheless, this real-time sequence has not been validated against a standard SSFP acquisition in a large clinical cohort. This prospective study sought to compare LV volumetric and wall motion assessment using both techniques in patients with known or suspected cardiac disease.

## Methods

We prospectively recruited 205 adult patients undergoing clinical CMR at a single tertiary cardiac centre (Glenfield Hospital, Leicester, UK). The study was approved by the UK National Research Ethics Service (REC reference 20/SC/0286) and registered on ClinicalTrials.gov (NCT05221853). The study was conducted in accordance with the Declaration of Helsinki, under the oversight of the University of Leicester.

### Study protocol

Consecutive patients referred for clinical CMR were studied at 3-Tesla (Magnetom Vida or Skyra, Siemens Healthineers, Erlangen, Germany). Functional cine images were acquired by 1) standard SSFP and 2) real-time cine sequences in a randomized order following administration of a gadoterate-based contrast agent (0.075–0.15 mmol/kg). A breath-hold, ECG-triggered SSFP pulse sequence was used to acquire a contiguous SAX stack [typical sequence parameters: slice thickness 8 mm, distance factor 25%, repetition time (TR) 42 ms, echo time (TE) 1.5 ms, matrix 256 × 204, field of view (FOV) 360 mm, FOV phase 81.3%, flip angle 45°]. In addition, patients underwent a free-breathing, multi-slice, ECG-triggered, real-time cine sequence, which acquired an entire SAX stack in approximately 1 min (typical sequence parameters: slice thickness 8 mm, distance factor 25%, TR 43 ms, TE 1.1 ms, matrix 160 × 92, FOV 360 mm, FOV phase 75%, flip angle 45°).^10^ For real-time imaging, the acquisition window was 4000 ms per slice (acquired over multiple R-R intervals) providing 93 phases for the raw image series. Automated reconstruction with the Gadgetron software framework outputted a retro-gated series selecting one heartbeat from each slice (closest to median R-R interval for all beats) with 30 calculated phases using temporal interpolation, voxel size 2.9 × 2.3 × 8 mm (interpolated to 2.3 × 2.3 mm).^13^

### Image analysis

#### Volumetric and wall thickness assessment

CMR cine image dataset pairs were split and coded with separate random identifiers to ensure blinding between sequences. Images were analysed offline, in a random order and blinded to clinical details using certified software (cvi42, v5.10.1, Circle Cardiovascular Imaging, Calgary, Canada). Image quality was graded on a 4-point scale: 0 = not analysable, 1 = moderate, artefact present but images still analysable, 2 = good, some artefact present but not in the region of interest, and 3 = excellent. For the real-time sequence, the reconstructed SAX images with 30 phases were analysed alongside the corresponding ECG to confirm cardiac cycle reconstruction was not erroneously triggered by a T-wave and each slice maintained a consistent R-R interval. Endocardial and epicardial contours were manually drawn for the LV in end-diastolic and end-systolic phases of each slice, excluding trabeculations and papillary muscles. The end-diastolic volume (EDV), end-systolic volume (ESV), stroke volume (SV), left ventricular mass (LVM), and ejection fraction (EF) were calculated as previously recommended.^14^ Maximal wall thickness (MWT) was determined automatically in the end-diastolic phase for each patient.

#### Wall motion assessment

Qualitative resting wall motion assessment was performed using the 16-segment American Heart Association model^15^ using standard segmental scoring: 1 = normal, 2 = hypokinetic, 3 = akinetic, 4 = dyskinetic/aneurysmal. A wall motion score index (WMSi) was derived for each patient by summing the segmental wall motion score and dividing by total myocardial segments.^16^

### Statistical analysis

Continuous data are expressed as mean ± standard deviation. Categorical data are presented as absolute value (%). Paired sample *t*-tests were used to compare within group differences. Intraclass correlation coefficient (ICC) with (95% confidence interval) were used to determine agreement in volumetric, wall motion, and thickness assessment between sequences and visualized using Bland–Altman plots.^17^ Inter-observer variability of volumetric assessment was judged by a second observer in a subset of 20 paired scans. Intra-observer variability was assessed by a single observer re-analysing a subset of 20 paired scans, blinded to previous results and following an interval of 4 weeks.

### Sample size calculation

A sample size of 198 participants achieves 90% power at the 2.5% significance level in an equivalence test of the mean difference in EF, using a paired study design with two one-sided *t*-tests. This is based on equivalence limits being −1% and 1%, and the actual mean of paired differences being zero, with a standard deviation of 3.9 (estimated from pilot data.^18^)

## Results

Two hundred and two patients (mean age 61 ± 14 years, 51% male, 14% irregular rhythm) were included in the final analysis. Three participants were excluded due to failed automated reconstruction of the real-time images (due to inconsistent ECG triggering within the captured raw image series). Baseline characteristics and study indications are presented in *Table 1*. Predominantly, CMR was undertaken for functional, ischaemia, or viability assessment.

**Table 1:** Baseline characteristics and clinical indications for CMR

**Demographics**	
Age (years)	61 ± 14
Male	103 (51%)
BMI (kg/m^2^)	29 ± 7
**Clinical indications for CMR**	
Volumetric assessment	77 (38%)
Ischaemia assessment	95 (47%)
Viability assessment	24 (12%)
Identification of mass	1 (<1%)
Pericardial disease	2 (1%)
Valvulopathy	2 (1%)
Aortopathy	1 (<1%)

Data are presented as mean ± SD or absolute value (%).

### Image quality

**Figure 1:**
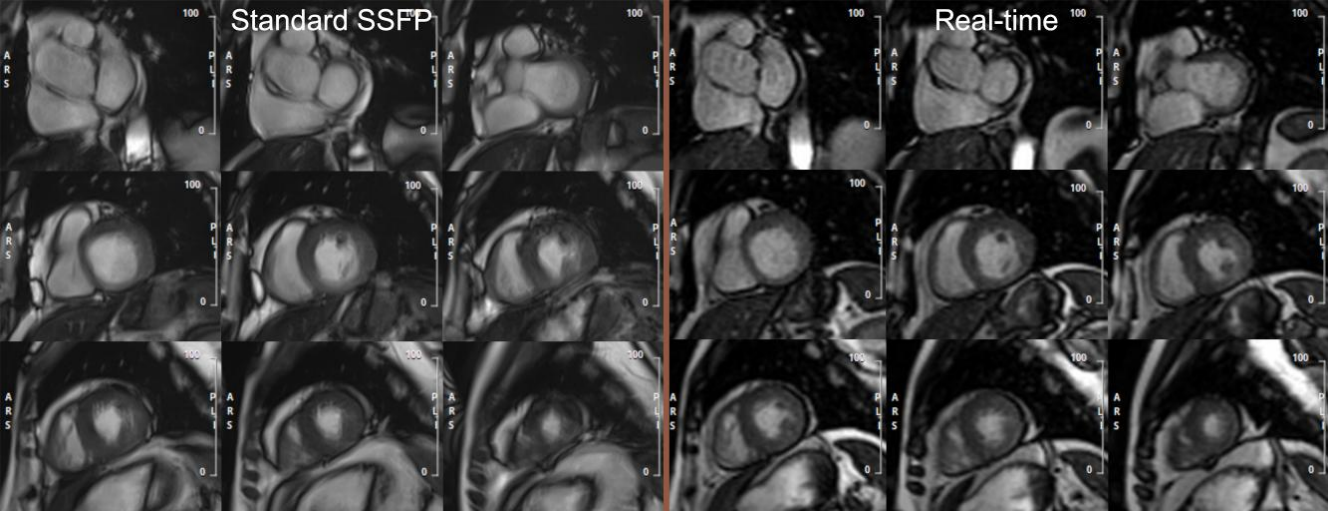
Comparison of image quality between SSFP and real-time imaging in a patient with the inability to perform repeated breath-holds.

All real-time and standard SSFP cine acquisitions were analysable. Real-time images demonstrated ‘excellent’ image quality in 76% and ‘good’ in 17% of cases. In those with an irregular rhythm (*n* = 28), real-time imaging was rated as ‘good’ or ‘excellent’ in 89% of cases *Table 2* and *Figure 1*.

**Table 2:** Comparison of image quality between SSFP and real-time cine imaging

	Standard SSFP	Real-time
**All patients (*n* = 202)**		
Excellent	104 (51%)	154 (76%)
Good	69 (34%)	35 (17%)
Moderate	29 (14%)	13 (6%)
Unanalysable	0	0
**Irregular rhythm (*n* = 28)**		
Excellent	6 (21%)	21 (75%)
Good	11 (39%)	4 (14%)
Moderate	11 (39%)	3 (11%)
Unanalysable	0	0

### Volumetric and functional assessment

**Figure 2:**
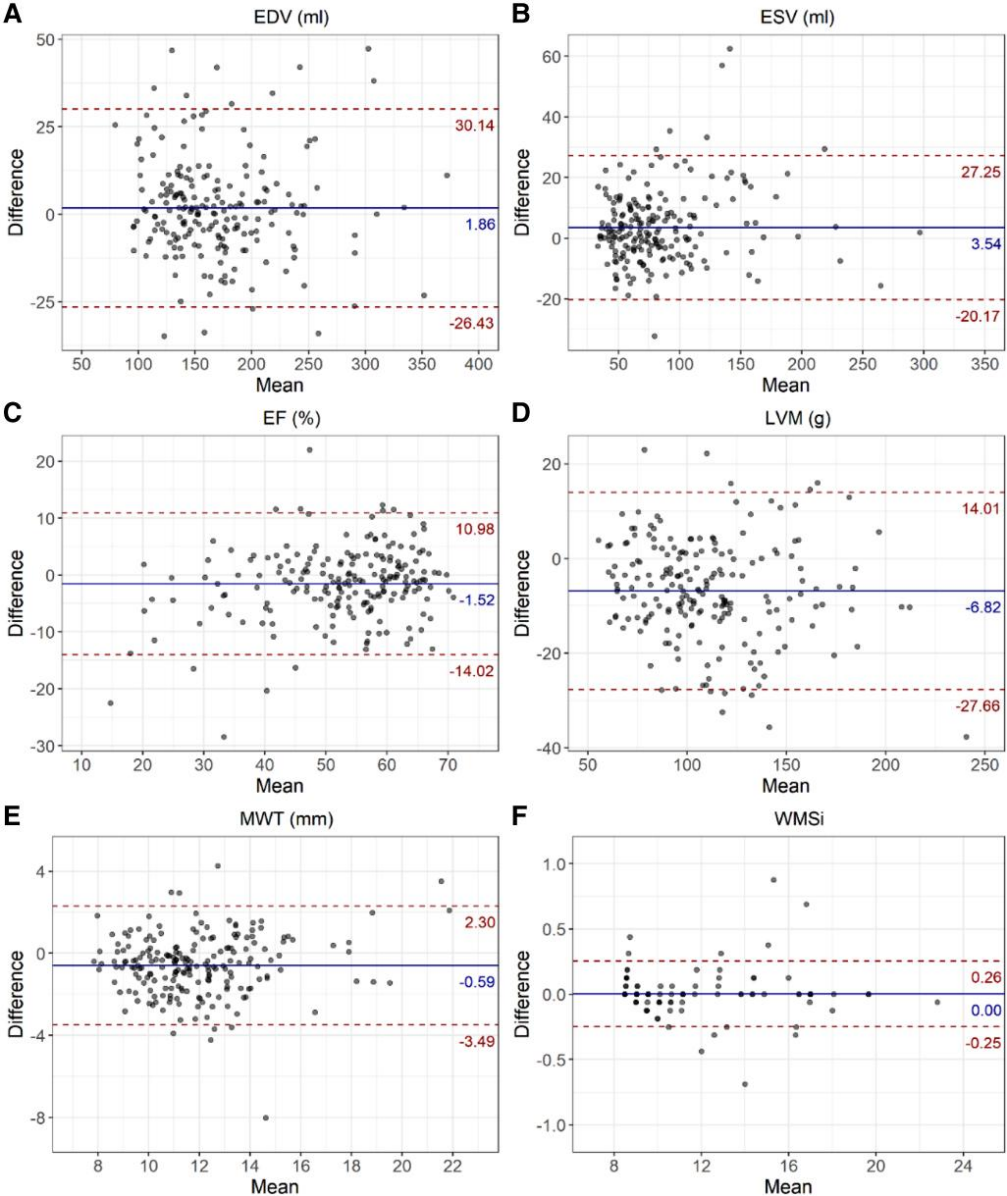
Bland–Altman plots illustrating the agreement between the real-time and standard SSFP cine sequences. *A*) EDV, *B*) ESV, *C*) EF, *D*) LVM, *E*) MWT, and *F*) WMSi. Each plot represents the difference between measurements plotted against their mean, with solid blue lines denoting the mean difference and red dashed lines indicating ±1.96 standard deviations from the mean difference.

There was no significant difference in EDV between real-time and standard SSFP cines (170.5 ± 51.0 mL vs. 168.7 ± 51.7 mL, *P* = 0.069). However, ESV was higher with real-time than with standard SSFP (85.3 ± 44.1 mL vs. 81.8 ± 42.4 mL, *P* < 0.001) with a correspondingly lower EF (51.7 ± 12.6% vs. 53.2 ± 11.2%, *P* < 0.001). Furthermore, LVM was lower with real-time compared with standard SSFP (107.4 ± 32.9 g vs. 114.3 ± 34.3 g, *P* < 0.001). All LV volumetric assessments showed good-excellent agreement between the two methods [EDV: ICC 0.96 (0.95–0.97), ESV: 0.96 (0.94–0.97), EF: 0.85 (0.81–0.88) and LVM: 0.93 (0.91–0.95); *Table 3* and *Figure 2*).

**Table 3:** Left ventricular volumetric, mass, maximal wall thickness, and wall motion score index assessment by real-time and standard SSFP cine imaging

	Real-time	Standard SSFP	Mean Difference (95% CI)	*P*-value	ICC (95% CI)
**EDV (mL)**	170.5 ± 51.0	168.7 ± 51.7	1.86 (−0.14, 3.86)	0.069	0.96 (0.95, 0.97)
**ESV (mL)**	85.3 ± 44.1	81.8 ± 42.4	3.54 (1.87, 5.22)	<0.001	0.96 (0.94, 0.97)
**EF (%)**	51.7 ± 12.6	53.2 ± 11.2	−1.52 (−2.41, −0.64)	0.001	0.85 (0.81, 0.88)
**LVM (g)**	107.4 ± 32.9	114.3 ± 34.3	−6.82 (−8.30, −5.35)	<0.001	0.93 (0.91, 0.95)
**MWT (mm)**	11.6 ± 2.6	12.2 ± 2.4	−0.59 (−0.80, −0.39)	<0.001	0.80 (0.75, 0.85)
**WMSi**	1.2 ± 0.4	1.2 ± 0.3	0.00 (−0.01, 0.02)	0.632	0.93 (0.91, 0.95)

CI, confidence interval; EDV, end-diastolic volume; EF, ejection fraction; ESV, end-systolic volume; LVM, left ventricular mass; MWT, maximal wall thickness; WMSi, wall motion score index.

Data compared using paired student *t*-test.

### Ejection fraction categorization

With standard SSFP as the reference standard, real-time imaging had a high discriminatory ability to correctly classify severe LV systolic dysfunction (sensitivity 82% and specificity 97% for EF ≤30% and 94% and 97% for ≤35%, respectively) and normal LV systolic function (sensitivity 88% and specificity 95% for EF >50%). In patients with impaired LV systolic function (EF 31–40% and 41–50%), the sensitivity of real-time to correctly classify EF was moderate (56% and 63%, respectively) with high specificity (95% and 89%, respectively; *Table 4*). *Table 5* compares the categorization of EF between real-time and SSFP imaging.

**Table 4:** Diagnostic performance of real-time imaging to classify ejection fraction

EF (%)	Sensitivity	Specificity	Diagnostic accuracy
≤35	94.1%	96.8%	96.5%
≤30	81.8%	97.4%	96.5%
31–40	55.6%	94.5%	91.1%
41–50	62.9%	89.2%	84.7%
>50	88.4%	95.3%	91.7%

Standard SSFP imaging as the reference standard.

**Table 5:** Comparison between real-time and SSFP cine imaging ejection fraction categorisation

	Real-time					
SSFP	EF (%)	≤30	31–40	41–50	>50	Total
	≤30	9	2	0	0	11
	31–40	3	10	3	2	18
	41–50	2	7	22	4	35
	>50	0	1	15	122	138
	Total	14	20	40	128	202

### Maximal wall thickness

MWT was lower with real-time compared with standard SSFP (11.6 ± 2.6 mm vs. 12.2 ± 2.4 mm, *P* < 0.001). There was good agreement between both techniques: ICC 0.80 (0.75–0.85; *Table 3*).

### Wall motion score

There was no significant difference in WMSi between real-time and standard SSFP (1.18 ± 0.35 vs. 1.17 ± 0.33, *P* = 0.632). Intraclass correlation coefficient revealed excellent agreement between techniques: 0.93 (0.91–0.95; *Table 3*).

### Impact of arrhythmia

Twenty-eight patients had evidence of arrhythmia. Consistent with the entire cohort, there were no significant differences in EDV or WMSi between real-time and standard SSFP sequences. However, ESV was higher with real-time, leading to a corresponding reduction in EF. Intraclass correlation coefficients revealed excellent agreement for EDV, ESV, LVM, and WMSi and good reliability for EF between sequences (*Table 6*).

**Table 6:** LV volumetric and functional assessment by real-time and standard SSFP cine imaging in patients with arrhythmia

	Real-time	Standard SSFP	Mean Difference (95% CI)	*P*-value	ICC (95% CI)
EDV (mL)	166.1 ± 53.7	163.8 ± 57.2	2.36 (−4.44, 9.16)	0.482	0.95 (0.90, 0.98)
ESV (mL)	98.7 ± 54.8	91.3 ± 51.3	7.43 (1.33, 13.53)	0.019	0.95 (0.89, 0.98)
EF (%)	43.7 ± 16.6	47.4 ± 14.1	−3.71 (−7.14, −0.27)	0.036	0.81 (0.64, 0.91)
LVM (g)	116.0 ± 41.5	119.4 ± 41.9	−3.41 (−8.30, 1.48)	0.164	0.94 (0.87, 0.97)
MWT (mm)	12.9 ± 3.6	12.9 ± 3.1	0.05 (−0.55, 0.66)	0.857	0.90 (0.79, 0.95)
WMSi	1.3 ± 0.5	1.3 ± 0.4	0.00 (−0.09, 0.09)	0.999	0.88 (0.75, 0.94)

EDV, end-diastolic volume; EF, ejection fraction; ESV, end-systolic volume; ICC, intraclass correlation coefficient; LV, left ventricular; LVM, left ventricular mass; MWT, maximal wall thickness; WMSi, wall motion score index.

Within group differences compared with paired student *t*-test.

### Inter- and intra-observer variability

For analysis of real-time cine images, there was excellent agreement across all LV parameters between observers (ICC 0.96–0.99). Similarly, there was excellent intra-observer agreement across all real-time cine LV parameters (ICC 0.94–0.99; *Table 7*).

**Table 7:** Inter- and intra-observer variability of LV assessment by real-time cine imaging

	Inter-observer variability ICC (95% CI)	Intra-observer variability ICC (95% CI)
EDV (mL)	0.992 (0.979, 0.997)	0.991 (0.977, 0.996)
ESV (mL)	0.994 (0.986, 0.998)	0.986 (0.966, 0.994)
EF (%)	0.981 (0.953, 0.992)	0.967 (0.919, 0.987)
LVM (g)	0.958 (0.898, 0.983)	0.939 (0.855, 0.975)

EDV, end-diastolic volume; EF, ejection fraction; ESV, end-systolic volume; ICC, Intraclass correlation coefficient; LV, left ventricle; LVM, left ventricular mass.

## Discussion

To our knowledge, this is the largest randomized prospective study to evaluate the reproducibility of any real-time cine sequence for LV volumetric assessment and the first to compare wall thickness and motion assessment between the two imaging methods. Real-time acquisitions demonstrated good-excellent reproducibility of LV volumetric, maximal wall thickness, and resting wall motion analysis compared with a standard SSFP sequence. This rapid imaging method may be readily integrated into clinical practice, especially in situations that require shorter scan times (patients with the inability to lie flat for prolonged periods or those with arrhythmia).

Previous work includes a single-centre study of 50 participants (25 healthy volunteers and 25 patients with structurally normal hearts) with comparison of EDV, ESV, and LVM using real-time and standard SSFP cine imaging. Results showed minimal bias (+1.1 mL for EDV, + 5.7 mL for ESV and +0.8 g for LVM) and excellent correlations for EDV, ESV and LVM (r = 0.98, 0.95 and 0.99, respectively).^19^ Despite strong agreement between both techniques, the limited sample size of younger adults primarily in sinus rhythm may not fully reflect the clinical populations that could benefit from rapid, free-breathing cine imaging.

Another study evaluated the feasibility and accuracy of a single breath-hold real-time sequence with incoherent k-space undersampling and iterative reconstruction in 41 patients with non-ischemic cardiomyopathy (41 ± 17 years, 37% male).^20^ Real-time imaging underestimated LV volumes and mass compared with a standard SSFP sequence but showed strong correlations (EDV r = 0.98, ESV r = 0.99, SV r = 0.89, EF r = 0.93 and LVM r = 0.92). The study, however, was limited to patients in sinus rhythm. Similar to our findings, there was an underestimation of LV mass by real-time imaging. However, given the sequence we utilized was free-breathing, there will be inherent variability in the position of the heart during acquisition, which can impact the orientation of imaging slices relative to the LV, alongside the differences in the extent of susceptibility artefact at the heart–lung interface. These factors may have led to variations in the contouring of the epicardial border and hence, differences in LV mass.

Another study compared compressed sensing (CS) real-time cine imaging with standard SSFP for quantifying LV volumes in 81 subjects, finding no significant differences and good correlations across all measurements.^21^ However, only patients in sinus rhythm were studied and those who were unable to hold their breath were excluded, precluding precise conclusions to be drawn. A similar study in 100 patients with cardiac disease demonstrated excellent correlations between CS real-time and standard SSFP imaging for LVEF, LVEDV, and LVM (*r* > 0.99, *r* > 0.99, and *r* = 0.98, respectively).^22^ Despite a shorter scan time with this real-time sequence, the data reconstruction was time-consuming (requiring up to 30 min) and thus would prevent integration within the clinical environment.

In contrast to previous studies, which involved smaller, selected cohorts in sinus rhythm with normal LV systolic function, our study included 202 consecutive patients with known or suspected cardiac disease referred for clinical CMR, and included those with arrhythmia. A major strength to real-time imaging is its speed of acquisition: whereas a standard SSFP SAX stack can take between 5 and 8 min, a real-time SAX stack can be acquired and automatically reconstructed within 1 min. This has the potential to refine clinical workflows, shortening scan times and maximizing throughput, as well as improving tolerability for patients.

Moreover, we demonstrated good–excellent agreement between real-time cine and standard SSFP for all parameters of LV volumetric assessments (ICC 0.85–0.96). However, in keeping with the published literature,^19^ real-time cine imaging exhibited a tendency to overestimate ESV, leading to a subsequent underestimation of EF. This may be due to the variability in SV at end-inspiration and end-expiration, which may differ between breath-hold and free-breathing sequences, whereby the latter captures respiratory variations in LV volumetry whereas a traditional breath-hold technique may not.^23^ Therefore, real-time cine sequences may provide more physiological assessment of LV volumetry. Despite this, the degree of biases in ESV and LVM was minimal and therefore unlikely to be clinically significant.

In our study, real-time imaging had a high diagnostic performance to correctly identify those with normal LV systolic function or severe LV systolic dysfunction (EF ≤35%—a widely recommended threshold for the initiation of drug and/or device therapy).^24^ This underscores the potential role of real-time imaging in the clinical arena to reliably guide therapeutic decisions.

## Limitations

This single-centre study included a relatively small number of patients with arrhythmia (14%) and all scans were performed at 3-Tesla. Furthermore, inter and intra-observer variability was only assessed in a subset of patients (10%) and inter-observer agreement only involved one additional reviewer. Nonetheless, this was a prospective, randomized, blinded study with a large cohort of patients, which mitigates some of the biases seen in other studies and is reflective of real-world clinical practice.

## Conclusion

Free-breathing real-time cine imaging is a reliable alternative to segmented multi-breath-hold SSFP for the assessment of LV volumetric, wall thickness and resting wall motion analysis. This accelerated imaging technique can be readily integrated into clinical practice, improving the tolerability and efficiency of CMR, particularly in situations which demand shortened examination times, whilst proving robust in patients with cardiac arrhythmia or dyspnoea.

## Data Availability

The data underlying this article will be shared on reasonable request to the corresponding author.

## Lead author biography

Dr Mohamed Elshibly is a UK-based specialist registrar in interventional cardiology with a strong focus on cardiovascular research. He is currently pursuing a PhD at the University of Leicester, with his work centred on advancing the understanding of myocardial perfusion and coronary artery disease. Dr Elshibly has particular expertise in cardiovascular magnetic resonance imaging and has contributed to several key research initiatives aimed at enhancing diagnostic accuracy and improving patient outcomes. Actively involved in clinical trials and academic collaborations, he is committed to translating research findings into real-world applications that benefit patients with cardiovascular disease.
